# A Case Report of Coronary Arteriovenous Fistulas with an Unruptured Coronary Artery Aneurysm Successfully Treated by Surgery

**DOI:** 10.1155/2012/314685

**Published:** 2012-11-13

**Authors:** Nobuhiro Takeuchi, Masanori Takada, Yoshiharu Nishibori, Takao Maruyama

**Affiliations:** Division of Cardiology, Kawasaki Hospital, Kobe, Japan

## Abstract

A 58-year-old female with a history of Wolff-Parkinson-White syndrome presented at our institution with palpitations and chest pain. Electrocardiography revealed paroxysmal supraventricular tachycardia with a heart rate of 188 beats/min. Antiarrhythmic drugs were ineffective, and tachycardia was resolved by electrical cardioversion. Transthoracic echocardiography revealed abnormal vessels around the right coronary artery (RCA) and pulmonary artery (PA); in addition, we suspected coronary arteriovenous fistula (CAVF). Coronary angiography and coronary computed tomography revealed dilated fistula vessels, with a 1 cm saccular aneurysm around the RCA, originating from the proximal RCA and left anterior descending artery into the main trunk of PA. Therefore, we confirmed the diagnosis of CAVF with an unruptured aneurysm. We surgically ligated and clipped the fistula vessels and resected the aneurysm. The resected aneurysm measured 1 × 1 cm in size. Pathological examination of the resected aneurysm revealed hypertrophic walls comprising proliferating fibroblasts cells thin elastic fibers. Very few atherosclerotic changes manifested in the aneurysm walls. We report the case of a patient with CAVF and an unruptured coronary artery aneurysm who was successfully treated by surgery.

## 1. Introduction

Coronary arteriovenous fistula (CAVF) is a rare congenital heart disease. In recent years, the widespread use of coronary angiography (CAG) has led to increased rate of detection of CAVF. In approximately 20% of patients, CAVF is accompanied with coronary artery aneurysm (CAA) [1]. Here we report the case of a patient with CAVF and an unruptured CAA who underwent successful surgical treatment.

## 2. Case Presentation

**Figure 1:**
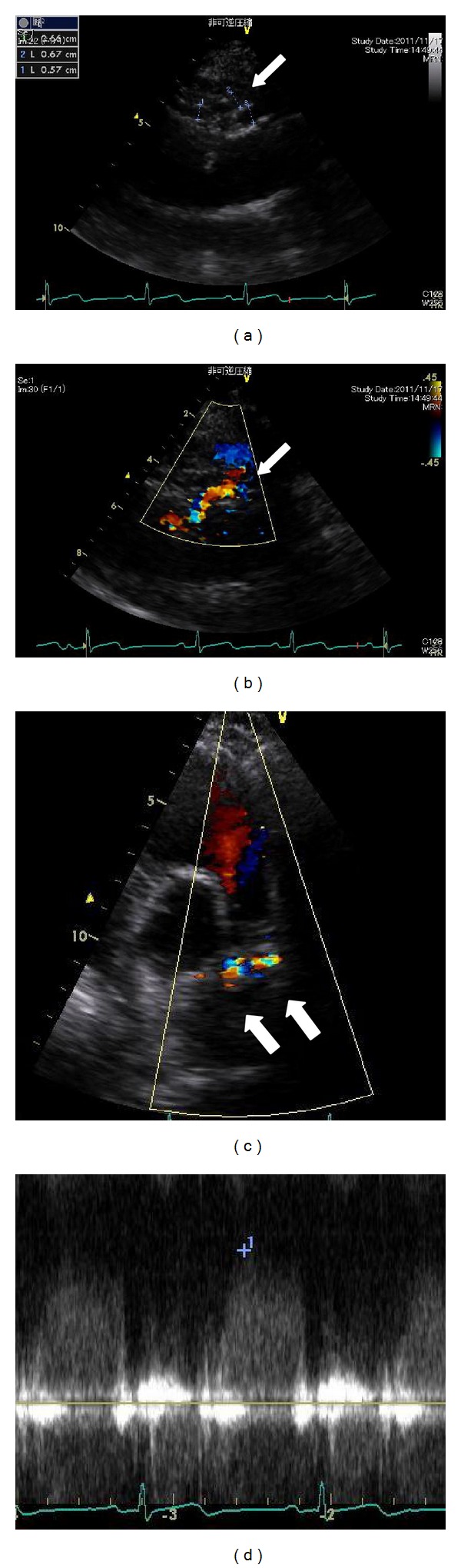
Transthoracic echocardiography. Abnormal, tortuous vessels were observed around the right coronary artery, and flow signals were detected by Doppler color flow imaging (a), (b). Abnormal vessels, predominantly exhibiting diastolic flow signals, were observed around the pulmonary trunk (c), (d).

**Figure 2:**
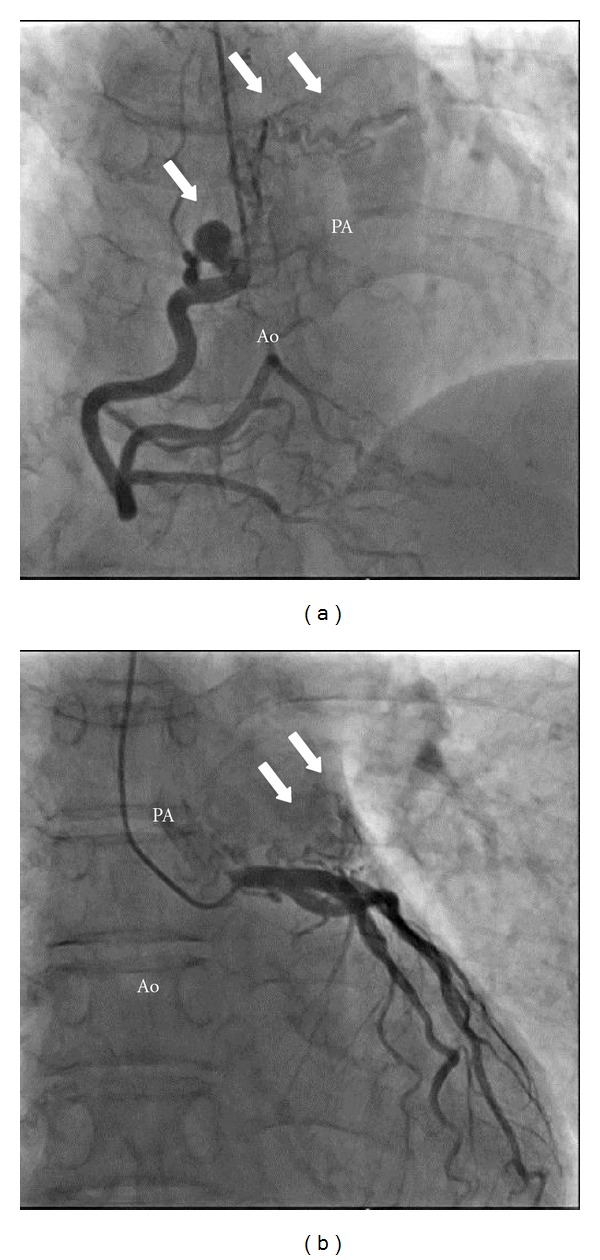
Coronary angiography. Fistula vessels (arrows) with a saccular aneurysm were observed originating from the proximal right coronary artery and joined the pulmonary artery (PA) (a). Fistula vessels (arrows) originating from the proximal left anterior descending artery joined the PA (b).

**Figure 3:**
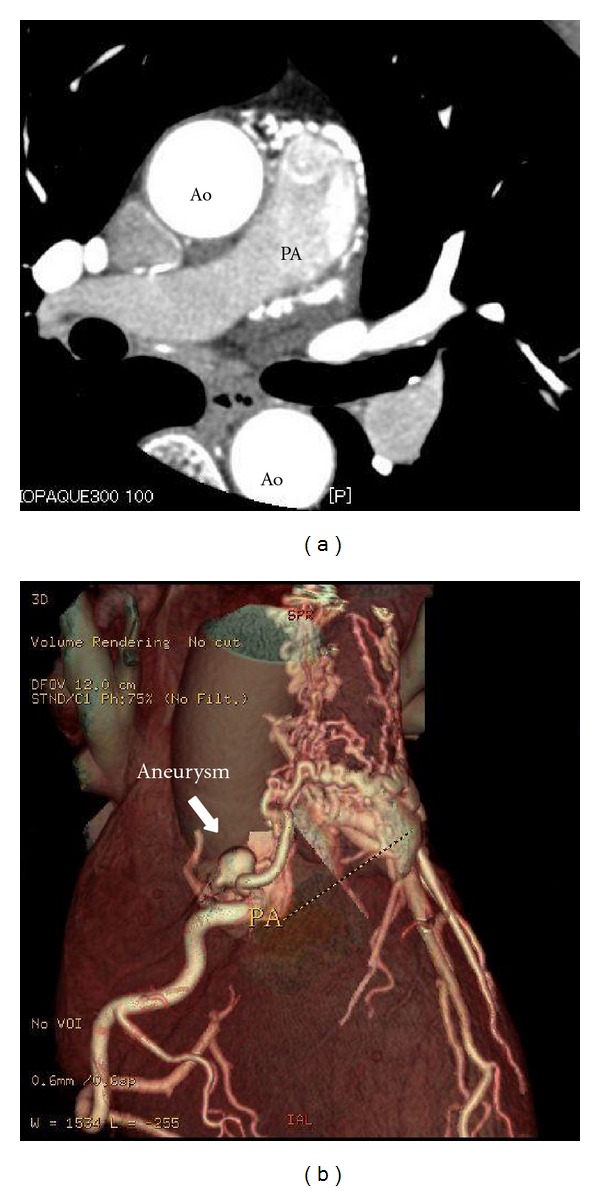
Coronary computed tomography. An axial section of the computed tomography (CT) image revealed dilated vessels around the pulmonary artery PA (a). Three-dimensional CT revealed a saccular aneurysm (arrow), 1 cm in diameter, around the right coronary artery (RCA), and fistula vessels originating from the proximal RCA and left anterior descending artery joined the pulmonary trunk (b).

**Figure 4:**
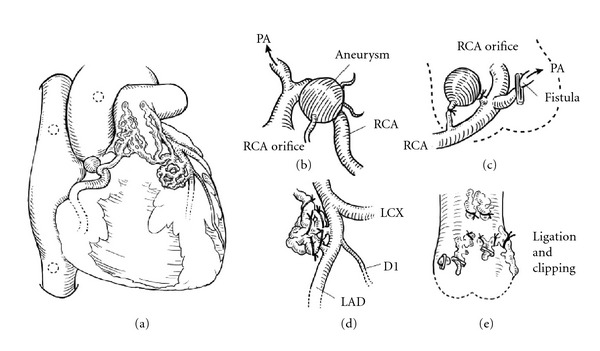
Operative findings. A coronary artery aneurysm was detected around the right coronary artery (RCA) and fistula vessels originating from the proximal RCA and left anterior descending artery (LAD) into the pulmonary trunk (a). The aneurysm was ligated and resected, and the fistula vessels were clipped (b), (c). Fistula vessels from the LAD were ligated (d), and fistula vessels around the pulmonary trunk were ligated and clipped (e).

**Figure 5:**
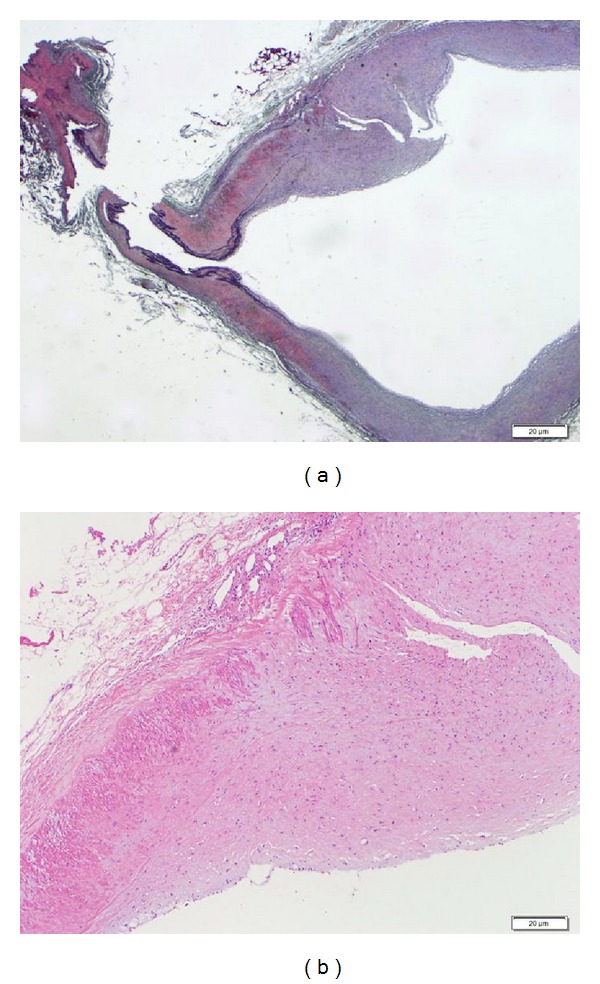
Pathological findings. The resected aneurysm revealed hypertrophic walls comprising proliferating fibroblasts covered with thin elastic fibers (a), (b). Few atherosclerotic changes were evident in the aneurysm walls. (a) Elastica van Gieson stain; (b) hematoxylin and eosin stain.

A 58-year-old female with a history of Wolff-Parkinson-White syndrome presented at our institution with palpitations and chest pain. Her systolic blood pressure was 80 mm Hg, and her heart rate was 188 beats/min. Her chest was clear on auscultation, and no abnormal murmur could be heard. In addition, blood chemistry findings including cardiac enzyme and lipid profiles were within normal limits. Electrocardiography revealed paroxysmal supraventricular tachycardia; however, chest radiograph did not present any signs of cardiomegaly or pulmonary congestion. Antiarrhythmic drugs, such as cibenzoline and adenosine, were ineffective. Tachycardia was resolved by electrical cardioversion. Transthoracic echocardiography (TTE) revealed normal left ventricular systolic function (ejection fraction: 73%). However, we evaluated the parasternal long-axis view at the aortic valve level and observed abnormal, tortuous vessels around the right coronary artery (RCA) (Figures 1(a) and 1(b)). Doppler color flow imaging revealed predominantly diastolic flow signals (Figure 1(d)), close to the pulmonary trunk (Figure 1(c)). CAG (Figures 2(a) and 2(b)) and coronary computed tomography (CT) (Figures 3(a) and 3(b)) performed for further investigation revealed dilated fistula vessels originating from the proximal RCA and left anterior descending artery (LAD) and joining the pulmonary trunk; moreover, we detected a saccular aneurysm 1 cm in diameter, around the RCA. Subsequently, we confirmed the diagnosis of CAVF with an unruptured aneurysm and performed surgical treatment. Surgical findings (Figure 4(a)) concurred with the CAG and coronary CT findings. Fistula vessels between the proximal RCA and pulmonary trunk were ligated and clipped (Figures 4(b) and 4(c)); a saccular aneurysm, 1 cm in diameter around the proximal RCA, was resected. Fistula vessels between the proximal LAD and pulmonary artery (PA) were ligated and clipped (Figures 4(d) and 4(e)). Pathological examination of the resected aneurysm revealed hypertrophic walls comprising proliferating fibroblast cell covered with thin elastic fibers. Very few atherosclerotic changes were evident in the aneurysm walls (Figures 5(a) and 5(b)). The postoperative course was uneventful without complications, and the patient was discharged 17 days after surgery. 

## 3. Discussion

CAVF is defined as abnormal communications between the coronary arteries and cardiac chambers, great arteries, or vena cava. Although young patients are often asymptomatic, two-thirds of patients aged >20 years present clinical symptoms of heart failure, pulmonary hypertension, or angina pectoris [2, 3]. CAVF accounts for 14% of congenital coronary malformations [4] and is detected by chance in 0.1%-0.2% of CAG [5, 6]. In 1975, almost all the reported cases of CAVF were congenital, featuring communications between the RCA and right ventricles [7]. In recent years, the incidence of acquired CAVF has increased, as a complication of improved cardiovascular therapies, such as percutaneous transluminal coronary angioplasty [8], myocardial muscle biopsy [9], pacemaker lead placement [10], or open-heart surgery [11, 12]. 

During early embryonic cardiac development, the ventricular chambers are spongiform and consume oxygen and nutrition from the blood within the chambers. Communications between the coronary arteries and the cardiac chambers are thought to develop during coronary artery development. Approximately 55% of CAVF originates from the RCA; 35% originates from the left coronary artery; 10% originates from both these arteries. In addition, 41% of fistula inflow is from the right ventricle; 26% is from the right atrium; 17% is from the PA; 7% is from the coronary venous sinus; 3% is from the left ventricle; 1% is from the superior vena cava [13]. About 20% of CAVF is accompanied by CAA, and such cases of CAA are usually solitary [1]. Although CAVF itself is considered congenital, there are reports indicating other causes including atherosclerosis, inflammation, injury, or turbulent blood flow [14, 15]. 

Symptomatic CAVF is commonly indicated for surgery to prevent ventricular volume overloadto ventricular chambers [16–18]. The Konno criteria are commonly used as indications for surgery [19]:

- left-to-right shunt ratio >30%, regardless of symptoms;
- signs of ischemia or volume overload in the right ventricle on electrocardiography;
- progression of pulmonary hypertension or congestive heart failure;
- history of infective endocarditis;
- aneurysm formation in the coronary artery.

Some authors [20] propose that asymptomatic CAVF could be surgically treated to prevent the occurrence of coronary artery disease or infective endocarditis. Although spontaneous CAVF closure has been reported in pediatric cases [21], spontaneous closure is unlikely in adults. CAAs >3 cm in diameter are at the risk of rupture [22]. However, cases of rupture in a 1 cm CAA have been reported [23]; thus, CAVF with CAA of any size should be considered a surgical indication. The clinical symptom of CAVF includes heart failure, infective endocarditis, angina pectoris, fatigue, and respiratory infection. CAVF can present as angina pectoris or myocardial ischemia due to coronary steal syndrome. Aging, atherosclerosis, and increased resistance of the coronary arteries (CAs) lead to increased flow into the PA, causing myocardial ischemia. In addition, some studies report that CAVF itself may cause CA atherosclerosis.

Reports regarding surgical procedure for treatment of CAVF describe direct closure of fistulas from inside the PA through incision [24], ligation of fistulas from the epicardium [25], and closure by catheterization [26]. Selection of surgical method should be based on anatomic morphology of the inflow and outflow vessels resulting from CAVF. In cases where inflow vessels form a complex capillary network on the PA, closing the fistulas from inside the PA is indicated, as dealing with these fistulas from the epicardium could be challenging. In such cases, cardiopulmonary support is essential. In cases where inflow and outflow vessels can be clearly identified, ligating fistulas from outside the epicardium is indicated, without cardiopulmonary support. In our case, clear identification of fistula vessels was possible using coronary CT; thus, we opted to ligate fistula vessels from the epicardium. 

Coronary CT efficiently evaluates CA morphology. Although CAG poorly visualizes the outflow vessels of CAVF, it visualizes the inflow vessels of CAVF excellently. In addition, coronary CT is suited to evaluate communications between CAA and the vessels in CAVF with CAA. Coronary CT efficiently visualizes both the inflow and outflow vessels of CAA. Three-dimensional coronary CT imaging facilitates comprehension of the anatomic locations of the fistula, PA, aorta, and CAA before surgery and yields beneficial information that may help in selecting the appropriate surgical procedure. 

## 4. Conclusion

We report a case, where CAVF was suggested by TTE findings with the help of CAG and coronary CT. Coronary CT efficiently provided information on the anatomic location of the CAVF and CAA, along with anatomic morphology of fistula vessels; in addition, coronary CT was very useful for selection of the appropriate surgical method in this case.
